# TBK1 and Caspase-8 suppress necroptosis but activate NLRP3 inflammasome activation during CXCL4 and TLR8 costimulation

**DOI:** 10.1038/s41420-024-02280-0

**Published:** 2024-12-23

**Authors:** Ying Cui, Yaguang Zhang, Chao Yang

**Affiliations:** 1 Department of Blood Transfusion, The First Affiliated Hospital of Xi’an Jiaotong University, Xi’an, Shaan Xi China; 2 Med-X Institute, Center for Immunological and Metabolic Diseases, First Affiliated Hospital of Xi’an Jiaotong University, Xi’an Jiaotong University, Xi’an, China

**Keywords:** Cell death and immune response, Immune cell death

Both CXCL4 and TLR8 are co-existed in and associated with severities of multiple autoimmune diseases, such as systemic sclerosis, systemic lupus erythematosus and rheumatoid arthritis [1–4]. Thus, we hypothesized that both CXCL4 and TLR8 signaling crosstalk contributed to disease progression. Indeed, we recently demonstrated that CXCL4 and TLR8 costimulation drastically activated inflammatory gene expression and NLRP3 inflammasome activation in human monocytes [5, 6]. TBK1 and its related kinase IKKε play central roles in Toll-like receptors (TLRs) and DNA/RNA sensors to induce type I IFNs against virus and intracellular bacterial infection [7–9]. CXCL4 and TLR8 costimulation drastically activated TBK1, which in turn coupled with IRF5 to induce inflammatory gene expression and NLRP3 inflammasome activation [5, 6]. In line with the activation of TBK1, treatment of human primary monocytes with the combination of CXCL4 and the TLR8 ligand ORN8L induced a strong increase in CYLD phosphorylation (Fig. 1A), which is the downstream of TBK1 [10, 11]; and TBK1/IKKε can inhibit CYLD deubiquitinase activity by phosphorylation at serine 418 [11–13] (Fig. 1B). Indeed, TBK1/IKKε inhibitor MRT67307 could almost abolish (CXCL4 + TLR8) signaling-induced CYLD phosphorylation (Fig. 1C). CYLD activity is also regulated by Caspase-8-mediated cleavage [13, 14] (Fig. 1B). We observed that CYLD cleavage was increased with time after CXCL4 or TLR8 alone treatment while (CXCL4 + TLR8) costimulation significantly reduced CYLD cleavage (Fig. 1D). However, inhibition of TBK1 activation by MRT67307 increased CYLD cleavage in (CXCL4 + TLR8) signaling (Fig. 1E). The result was corroborated using two additional distinct TBK1/IKKε inhibitors (Fig. 1F). These data suggest that, in (CXCL4 + TLR8) costimulation, activation of TBK1 suppresses CYLD activation by phosphorylation, thereby reducing its cleavage by Caspase-8.

**Fig. 1:**
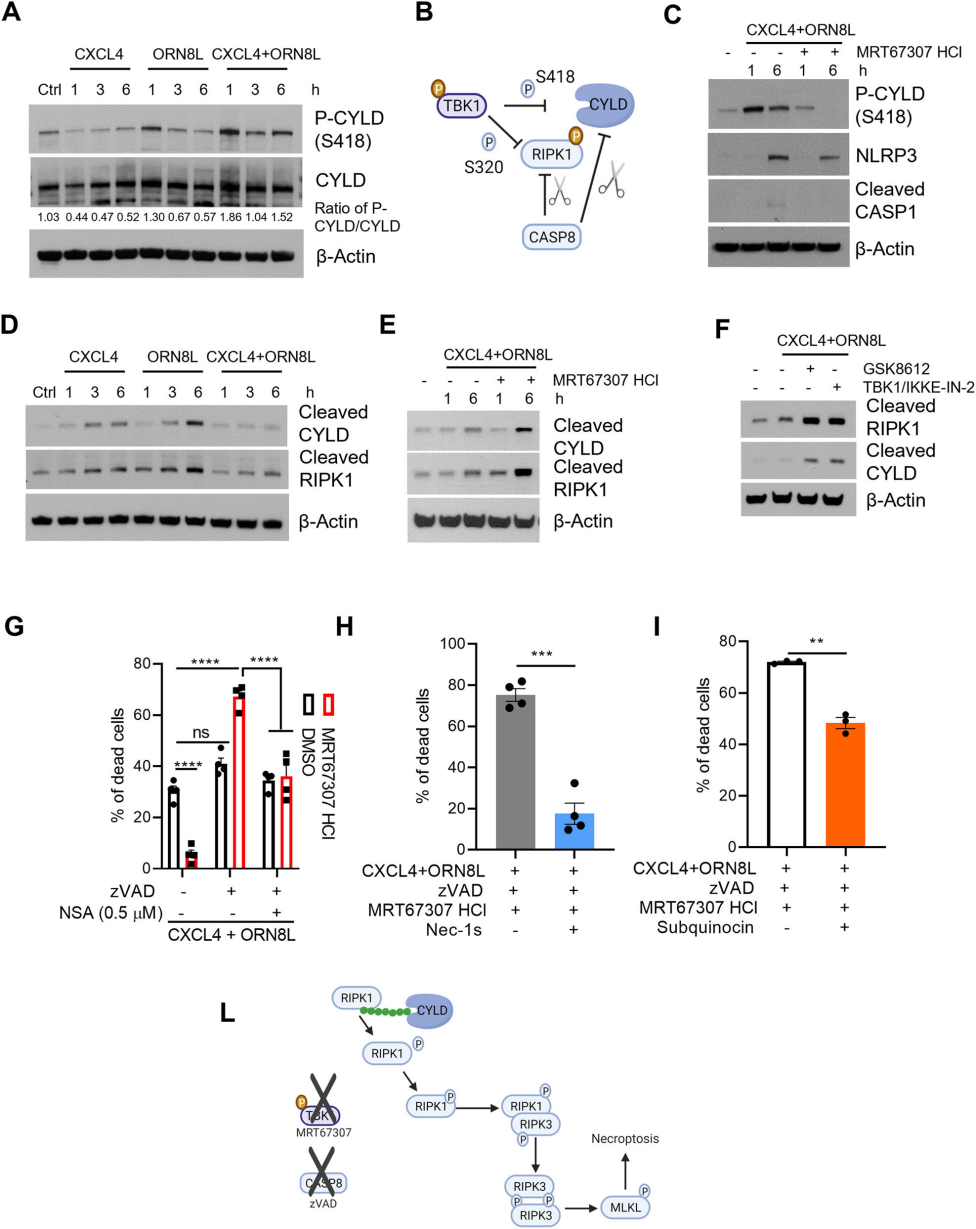
TBK1 regulates NLRP3 inflammasome activation and necroptosis in CXCL4 and TLR8 costimulation in human monocytes. **A**, **D** Immunoblots with whole cell lysates from monocytes stimulated with CXCL4 (10 μg/ml) and/or TLR8 ligand ORN8L (20 μg/ml) for indicated time-course. **B**, **L** Schematic diagrams. **C**–**F** Immunoblots of cleaved RIPK1 and CYLD, phospho-CYLD, NLRP3, phosphor-p65 and cleaved Caspase-1 (CASP1) with whole cell lysates from monocytes stimulated with CXCL4 + ORN8L in the presence of TBK1 inhibitors MRT67307 (10 μM) (**C**, **E**), or GSK8612 (50 μM) or TBK1/IKKε-IN-2 (1 μM) (**F**) for indicated time-course. **G**–**I** Flow cytometric analysis of cell viability in cells pre-treated with zVAD (50 µM), MRT67307, NSA, Nec-1s (30 µM) and/or Subquinocin (500 µM) for 30 min, and then stimulated with CXCL4 + ORN8L for 6 hr. The representative blots are from at least three independent experiments (**A**, **C**–**F**). Data is from 3 (**I**) or 4 (**G**, **I**) independent experiments. Data is depicted as mean ± SEM (**F**–**H**). ****p ≤ 0.0001; ***p ≤ 0.001; **p ≤ 0.01 by two-way ANOVA (**G**) or Paired t test (**H**, **I**).

RIPK1 kinase activity is regulated by TBK1 and Caspase-8 through direct phosphorylation at serine 320 and cleavage, respectively, while CYLD increases RIPK1 kinase activity by deubiquitylating K63 ubiquitin chain of RIPK1 [10, 15–17] (Fig. 1B). Similar to CYLD, RIPK1 cleavage was increased with time after CXCL4 or TLR8 signaling alone while no RIPK1 cleavage occurred in (CXCL4 + TLR8) signaling (Fig. 1D); inhibition of TBK1 increased RIPK1 cleavage during (CXCL4 + TLR8) stimulation (Fig. 1E, F). Thus, these data suggest that both TBK1 and Caspase-8 tightly suppress RIPK1 kinase activity during (CXCL4 + TLR8) stimulation; and this finding might explain why RIPK1 is not involved in (CXCL4 + TLR8)-induced cell death in the absence of Caspase-8 activity [6]. These data suggest that RIPK1 is tightly regulated in (CXCL4 + TLR8) costimulation. In our recent studies, we found that (CXCL4 + TLR8) induced NLRP3 inflammasome activation and Gasdermin D (GSDMD)-mediated pyroptosis and that TBK1 controlled NRLP3 expression and inhibition of TBK1 by inhibitor abolished inflammasome activation [5, 6] (Fig. 1C). In accord with the abolished NLRP3 inflammasome activation, inhibition of TBK1 also significantly reduced cell death in (CXCL4 + TLR8) costimulation (Fig. 1G). Intriguingly, blockade of Caspase-8 activation by Z-VAD-FMK (zVAD) did not increase much of cell death while additionally adding MRT67307 robustly increased (CXCL4 + TLR8)-induced cell death, which could be rescued by blocking necroptotic cell death mediator MLKL, RIPK1 and CYLD with their inhibitors Necrosulfonamide [6, 18] (NSA, 0.5 µM), Necrostatin 2 racemate (Nec-1s) and Subquinocin, respectively (Fig. 1H, I). Together, these findings suggest that TBK1 and Caspase-8 suppress necroptosis by tightly regulating CYLD and RIPK1 activity (Fig. 1L), and that TBK1 contributes to NLRP3 inflammasome activation and pyroptosis upon (CXCL4 + TLR8) costimulation.

Our studies describe a pathway that links the activation of TBK1 with NLRP3 expression and 2^nd^ signal-independent inflammasome activation and cell death in CXCL4 and TLR8 costimulation. We show that TBK1 activation tightly regulates CYLD activity and RIPK1 activation upon CXCL4 and TLR8 costimulation while CYLD and RIPK1 are regulated by Caspase-8-mediated cleavage in either CXCL4 or TLR8 alone [10, 13, 14, 17, 19]. Our recent study showed that TBK1 controls NLRP3 gene expression and inhibition of TBK1 activation abolished NLRP3-mediated inflammasome activation in CXCL4 and TLR8 costimulation, this is also true for (CXCL4 + TLR8) costimulation-induced cell death. Although inhibition of Caspase-8 activation suppresses NLRP3 inflammasome activation and GSDMD-mediated pyroptosis [6], the percentage of cell death did not reduce but slightly increased, suggesting that RIPK1 might keep intact and induce necroptosis to compensate the reduced pyroptosis in the absence of Caspase-8 activity [10, 17, 19]. Surprisingly, inhibition of both TBK1 and Caspase-8 activation significantly increases (CXCL4 + TLR8)-induced necroptosis, which could be suppressed by jointly inhibition of MLKL, RIPK1 or CYLD, respectively. These findings demonstrate that TBK1 and Caspase-8 act as double-check to tightly control CYLD and RIPK1-mediated necroptotic cytotoxicity in CXCL4 and TLR8 costimulation.

In summary, the activation of TBK1 cooperated with Caspase-8 act as gatekeepers to determine cell viability by suppressing CYLD and RIPK1 activities and by inducing NLRP3 inflammasome activation. These findings provide a new paradigm for the regulation of inflammasome activation and necroptosis that contribute to inflammatory disease pathogenesis.

## Supplementary information

Supplementary information
