# Supplementary material for: TBK1 and Caspase-8 suppress necroptosis but activate NLRP3 inflammasome activation during CXCL4 and TLR8 costimulation
Source: Cell Death Discov. 2024 Dec 23;10:511. doi: 10.1038/s41420-024-02280-0 (PMC11666742; doi:10.1038/s41420-024-02280-0)
Supplement: Supplementary file 1 — Supplementary information [file 41420_2024_2280_MOESM1_ESM.docx]

**TBK1 and Caspase-8 Suppress Necroptosis but Activate NLRP3 Inflammasome Activation during CXCL4 and TLR8 Costimulation**

Ying Cui^1^, Yaguang Zhang^2^, Chao Yang^1,2*^

^1^Department of Blood Transfusion, The First Affiliated Hospital of Xi'an Jiaotong University, 277 Yanta West Road, Yanta District, Xi'an, 710061, Shaan Xi, China;

^2^Med-X institute, Center for Immunological and Metabolic Diseases, First Affiliated Hospital of Xi'an Jiaotong University, Xi'an Jiaotong University, Xi’an, China.

^*^ Correspondence: ychmax@xjtufh.edu.cn

**Materials and Methods**

**Human cells**

Deidentified human buffy coats were purchased from Department of Blood Transfusion, The First Affiliated Hospital of Xi’an Jiaotong University following a protocol approved by The First Affiliated Hospital of Xi’an Jiaotong University Institutional Review Board. Peripheral blood mononuclear cells (PBMCs) were isolated with Lymphoprep (Accurate Chemical, Carle Place, NY, USA) via density gradient centrifugation and monocytes were purified from PBMCs with anti-CD14 magnetic beads as recommended by the manufacturer (Miltenyi Biotec). Monocytes were cultured overnight at 37^o^C, 5% CO_2_ in RPMI-1640 medium (Invitrogen) supplemented with 10% heat-inactivated defined FBS (HyClone Fisher), penicillin-streptomycin (Invitrogen), L-glutamine (Invitrogen) and 20 ng/ml human M-CSF. After at least 12 hr culture, the cells were treated as described in the figure legends and were harvested and prepared for total RNA extraction, protein extraction and flow cytometry.

**Flow cytometric analysis of cell death**

Single cell suspensions were stained with Fixable Viability Dye eFluor™ 780 (eBioscience, Cat. #65-0865-18) for 20 min at 4 ⁰C. Then, cells were washed with FACS buffer (PBS containing 2% calf serum and 1 mM EDTA) and were detected by flow cytometry. Dead cells were detected and excluded based on eFluor 780 fluorescence. Data were analyzed with FlowJo software.

**Western Blotting**

Cells were lysed in 50 µl of cold lysis buffer comprised of 50 mM Tris-HCl pH 7.4, 150 mM NaCl, 1 mM EDTA, 1% (vol/vol) Triton X-100, 2 mM Na3VO4, 1x phosSTOP EASYPACK, 1 mM Pefablock, and 1× EDTA-free complete protease inhibitor cocktail (Roche, Basel, Switzerland), and incubated for at least 10 min on ice. Then, cell debris was pelleted at top speed at 4^o^C for 5 min. The protein fraction in the supernatant was mixed with 4× Laemmli Sample buffer (BIO-RAD, Cat. #1610747) supplemented with 10% 2-mercroptoehanol (BME) (Sigma-Aldrich). Samples for Western blotting were subjected to electrophoresis on Bis-Tris gels (Invitrogen). Proteins were transferred to polyvinylidene difluoride membrane. Membranes were blocked in 5% (w/v) Bovine Serum Albumin in TBS (20 mM Tris, 50 mM NaCl, pH 8.0) with 0.1% (v/v) Tween 20 (TBST) at room temperature (RT) for at least 1 hr with shaking. Membranes were then incubated with target primary antibodies at 4 °C overnight with shaking and then washed 3 times in TBST for 10 min each time, then incubated with anti-mouse or anti-rabbit IgG secondary antibodies conjugated to horseradish peroxidase (HRP) (GE Healthcare, cat: NA9310V and NA9340V) diluted in TBST at room temperature for 1 hr with shaking. Next, membranes were washed 3 times in TBST at RT for with shaking. Antibody binding was detected using enhanced chemiluminescent substrates for HRP (ECL Western blotting reagents (PerkinElmer, cat: NEL105001EA)) or SuperSignal™ West Femto Maximum Sensitivity Substrate (Thermo Fisher Scientific, cat: 34095) according to the manufacturer's instructions, and visualized using premium autoradiography film (Thomas Scientific, cat: E3018). For membranes that required probing twice or more using different primary antibodies, RestoreTM PLUS Western blotting stripping buffer (Thermo Fisher Scientific) was applied on the blots with shaking for 15 - 20 min at RT following first time development. To detect multiple proteins on the same experimental filter, membranes were cut horizontally based on the molecular size of the target proteins. Antibodies used are identified in Supplementary Table.

**Statistical analysis**

Graphpad Prism for Windows was used for all statistical analysis. Information about the specific tests used, and the number of independent experiments is provided in figure legends. Two-way ANOVA with Sidak correction for multiple comparisons was used for grouped data. Otherwise, one-way ANOVA with the Geisser-Greenhouse correction and Tukey’s post hoc test for multiple comparisons was performed. For paired data, paired t test was used.

**Antibodies, TLR ligands, inhibitors, recombinant proteins and others:**

| **Antibodies Source CAS#** | | |
| --- | --- | --- |
| Phospho-CYLD (Ser418) Antibody | Cell Signaling Technology | 4500T |
| β-Actin (D6A8) Rabbit mAb | Cell Signaling Technology | 8457 |
| NLRP3 (D4D8T) Rabbit mAb | Cell Signaling Technology | 15101 |
| CYLD Antibody | Cell Signaling Technology | 4495S |
| RIP (D94C12) XP® Rabbit mAb | Abcam | 3493S |
| **TLR ligands, inhibitors, recombinant proteins** | | |
| ORN8L | Chemgenes Corporation |  |
| Recombination Human CXCL4 | PEPROTECH | 300-16 |
| PF-4 (CXCL4) human | Sigma-Aldrich | SRP3142 |
| MRT67307 HCl (dual IKKϵ and TBK1 inhibitor) | Selleckchem | S7948 |
| TBK1/IKKε-IN-2 | MCE | HY-12453 |
| GSK8612-TBK1 inhibitor | Selleckchem | S8872 |
| Necrosulfonamide (NSA) | Sigma-Aldrich | 480073 |
| Z-VAD-FMK-Caspase-8 inhibitor | R&D Systems | FMK001 |
| 7-Cl-O-Nec1 (ab221984)-RIPK1 inhibitor | abcam | ab221984 |
| Subquinocin (Novel inhibitor of CYLD) | Glixx Laboratories Inc | GLXC-22030 |
